# Development and evaluation of pH sensitive semi-interpenetrating networks: assessing the impact of itaconic acid and aloe vera on network swelling and cetirizine release

**DOI:** 10.3389/fbioe.2023.1173883

**Published:** 2023-05-09

**Authors:** Nyla Ajaz, Munnaza Bukhsh, Yousaf Kamal, Fauzia Rehman, Muhammad Irfan, Syed Haroon Khalid, Sajid Asghar, Waleed Y. Rizg, Sahar M. Bukhary, Khaled M. Hosny, Mohammed Alissa, Awaji Y. Safhi, Fahad Y. Sabei, Ikram Ullah Khan

**Affiliations:** ^1^ Department of Pharmaceutics, Faculty of Pharmaceutical Sciences, Government College University Faisalabad, Faisalabad, Pakistan; ^2^ Department of Pharmacy, The University of Faisalabad, Faisalabad, Pakistan; ^3^ Foundation University and Medical College Islamabad Department of Medicine, Islamabad, Pakistan; ^4^ Hamdard Institute of Pharmaceutical Sciences, Hamdard University Karachi, Islamabad Campus, Islamabad, Pakistan; ^5^ Center of Innovation in Personalized Medicine (CIPM), 3D Bioprinting Unit, King Abdulaziz University, Jeddah, Saudi Arabia; ^6^ Department of Pharmaceutics, Faculty of Pharmacy, King Abdulaziz University, Jeddah, Saudi Arabia; ^7^ Department of Chemical Laboratories, King Abdulaziz Medical City, Jeddah, Saudi Arabia; ^8^ Department of Medical Laboratory Sciences, College of Applied Medical Sciences, Prince Sattam Bin Abdulaziz University, Al-Kharj, Saudi Arabia; ^9^ Department of Pharmaceutics, College of Pharmacy, Jazan University, Jazan, Saudi Arabia

**Keywords:** semi-interpenetrating network, sustainability of natural resources, aloe vera, itaconic acid, pH sensitive

## Abstract

Hydrogels are crosslinked three-dimensional networks, and their properties can be easily tuned to target the various segments of the gastrointestinal tract (GIT). Cetirizine HCl (CTZ HCl) is an antihistaminic drug, which when given orally can upset the stomach. Moreover, this molecule has shown maximum absorption in the intestine. To address these issues, we developed a pH-responsive semi-interpenetrating polymer network (semi-IPN) for the delivery of CTZ HCl to the lower part of the GIT. Initially, 10 different formulations of itaconic acid-grafted-poly (acrylamide)/aloe vera [IA-g-poly (AAm)/aloe vera] semi-IPN were developed by varying the concentration of IA and aloe vera using the free radical polymerization technique. Based on swelling and sol-gel analysis, formulation F5 containing 0.3%w/w aloe vera and 6%w/w IA was chosen as the optimum formulation. The solid-state characterization of the optimized formulation (F5) revealed a successful incorporation of CTZ HCl in semi-IPN without any drug-destabilizing interaction. The *in vitro* drug release from F5 showed limited release in acidic media followed by a controlled release in the intestinal environment for over 72 h. Furthermore, during the *in vivo* evaluation, formulation F5 did not affect the hematological parameters, kidney, and liver functions. Clinical observations did not reveal any signs of illness in rabbits treated with hydrogels. Histopathological images of vital organs of treated animals showed normal cellular architecture. Thus, the results suggest a non-toxic nature and overall potential of the developed formulation as a targeted drug carrier.

## 1 Introduction

Drug carriers are intended for the safe and effective delivery of therapeutically active molecules to enhance their solubility, chemical stability, bioavailability, targetability, and pharmacological activity, as well as to reduce their side effects. In recent years, crosslinked polymeric carriers such as hydrogels have gained remarkable attention for the controlled and targeted delivery of the drug (Damiri et al., 2020). Hydrogels are three-dimensional networks where polymers are crosslinked via covalent bonding or are fastened together by intermolecular interactions. They possess a tremendous potential to hold or absorb large amounts of fluids within their interstitial spaces. However, homopolymer-based hydrogels have shown poor mechanical strength that leads to compromised structural integrity and burst release of entrapped molecules. Since 2000, scientists have been trying to overcome these limitations which can be easily overcome by using semi-IPN (Gong et al., 2003; Sedlačík et al., 2020). These networks are composed of two polymers, where the linear polymer penetrates the crosslinked network.

Polysaccharide-based hydrogels are widely engaged as drug carriers. According to existing literature, many natural polysaccharides (Bera et al., 2015) like aloe vera, starch, chitosan (Shalviri et al., 2010; Bajer et al., 2020), guar gum (George and Abraham, 2007), and Gum ghatti (Sharma et al., 2014a) have been used for the development of hydrogels. Aloe vera is a natural polymer that was used in the current study owing to ease of availability, biodegradability, economy, and biocompatibility. Several authors have highlighted the importance of aloe vera in different areas of the biomedical field owing to the presence of biologically active components. In tissue engineering, aloe vera has received considerable attention due to its biodegradability, biocompatibility, and low toxicity (Rahman et al., 2017). Aloe vera contains several amino acids, vitamins C and E, and zinc which play an important role in stimulating accelerated wound healing (Alven et al., 2021; Khan et al., 2023). It has also been investigated for the development of various drug carriers such as gels (Rompicherla et al., 2022), film (Hajian et al., 2017), sustained-release tablets (Laux et al., 2019), and microparticles (Pereira et al., 2014). It has been observed that blending aloe vera with other polymers not only increases their biocompatibility but also substantially improves the mechanical integrity of hydrogel-based matrices and films (Dey et al., 2015). Aloe vera is preferred over other polysaccharides owing to its high water-absorbing capacity and ease of modification/grafting due to the large number of functional groups in its backbone. Studies have demonstrated that the fluid-absorption capacity and mechanical strength of aloe vera can be further improved by grafting other monomers (Kumar et al., 2018).

Itaconic acid (IA) is an unsaturated dicarbonic acid where one carboxyl (COOH-) is linked with the ethylene group (Kumar et al., 2017). Acrylamide (Aam) is a white, colorless, and odorless monomer with good aqueous solubility (Faroon et al., 2009). Poly (AAm)-based hydrogels have been extensively studied owing to their excellent swelling capabilities (Jayaramudu et al., 2019), thermal stability (Zhang et al., 2022), and biocompatibility (Subramani et al., 2020). These properties are further improved by combining them with other polymers. The properties of polymeric blends or copolymers rely on the interactions between their components (Torres-Figueroa et al., 2020). IA was used to develop pH-sensitive hydrogels with many other monomers, e.g., poly (acrylic acid-co-IA) (Katime and Rodriguez, 2001) and poly (N-vinyl caprolactam-co-IA) (Cavus and Cakal, 2012). We came across other studies where AAm, IA, and other monomers were used in various combinations to develop pH-responsive hydrogels (Bera et al., 2015; Quintana et al., 2002; Katime et al., 2001; Caykara et al., 2004). Therefore, we aimed to formulate pH-sensitive semi-IPN hydrogels based on aloe vera, Aam, and IA for the site-specific delivery of Cetirizine HCl (CTZ HCl). Moreover, developed networks were extensively characterized by various *in vitro* and *in vivo* tests.

## 2 Materials and method

Aloe vera and acrylamide were purchased from Daejung reagents chemicals and metals Co., Ltd., Korea. Itaconic acid and cetirizine HCl were purchased from Shenyang polychemicals co., Ltd., China, and Remington Pharmaceutical Industries (Pvt) Ltd., Lahore, Pakistan, respectively. Ammonium persulfate and ethylene glycol dimethacrylate were procured from Sigma Aldrich. All other chemicals used were of analytical grade.

### 2.1 Development of IA-g-poly (AAm)/aloe vera

Different formulations of pH-sensitive semi-IPN hydrogels were prepared by using IA and aloe vera with a fixed concentration of crosslinkers (Table 1) via the free radical polymerization technique. In Brief, separate solutions of IA, Aam, and ammonium persulfate (APS) were prepared in deionized water with constant stirring at room temperature. To create free radicals, the initiator solution was added to the solution of aloe vera, while ethylene glycol dimethacrylate (EGDMA) was added to the AAm solution. Afterward, these two solutions were mixed, and IA was added dropwise at room temperature with continuous stirring to obtain a uniform solution. Subsequently, the resultant solution was sonicated (JP030S; Skymen Cleaning Equipment Shenzhen Co. Ltd., China) for 5 min to remove air bubbles. Then, the transparent solution was transferred to glass test tubes and deoxygenated with nitrogen gas. Tightly covered test tubes were placed in a pre-heated water bath maintained at 45°C to avoid bubble formation with a heating sequence at 45°C (2 hours), at 50°C (2 hours), at 55°C (2 hours), at 60°C (overnight), and further at 65°C (2 hours). Finally, the test tubes were removed from the water bath and were cooled at room temperature. Firm and cylindrical hydrogels were removed from the test tubes and cut into 8 mm discs with a sharp blade (Abbasi et al., 2019). Obtained hydrogel discs were washed with water and ethanol mixture (50:50). Finally, these discs were dried at room temperature and stored in a desiccator until further testing.

**TABLE 1:** The composition of IA-g-poly (AAm)/aloe vera semi-IPN hydrogels.

Code	Aloe vera (%w/w)	IA (%w/w)	AAm (%w/w)	EGDMA (%w/w)	APS (%w/w)
Alo1	0.07	6	20	0.40	0.30
Alo2	0.15	6	20	0.40	0.30
Alo3	0.30	6	20	0.40	0.30
Alo4	0.50	6	20	0.40	0.30
F1	0.30	0	20	0.40	0.30
F2	0.30	1	20	0.40	0.30
F3	0.30	2	20	0.40	0.30
F4	0.30	4	20	0.40	0.30
F5	0.30	6	20	0.40	0.30
F6	0.30	8	20	0.40	0.30

### 2.2 Swelling

For the optimization and determination of the pH-sensitive behavior of developed semi-IPN hydrogels, the swelling of blank and drug-loaded hydrogels was assessed by immersing the pre-weighed hydrogels in buffer solutions (pH 7.4 and pH 1.2). Hydrogels were removed at different time intervals, and the weights of swollen hydrogel discs were measured. The swelling index was calculated using the following formula (n = 3),

$$Swelling\;\left(\%\right)=\frac{\left(Wt-W0\right)}{W0}\times 100$$ (1)

“Wt” refers to the final weight of hydrogels at specified time intervals, and “Wo” refers to the initial weight of dried hydrogels (Wei et al., 2021).

### 2.3 Sol-gel fraction

To evaluate non-crosslinked polymeric content, sol-gel analysis was performed. Pre-weighed dried blank IA-g-poly (AAm)/aloe vera semi-IPN hydrogels were soaked in deionized water for 7 days with frequent shaking. Distilled water was replaced with fresh distilled water after 2 days. After 7 days, the swollen hydrogels were removed and dried at room temperature. The weight of the dried hydrogels was noted (Sohail et al., 2014). The following equations were employed to estimate the sol-gel percentage.

$$Sol\;fraction\;\left(\%\right)=\left(Wo-Wd\right)/Wo\times 100$$ (2)

$$Gel\;fraction\;\left(\%\right)=100-sol\;fraction\;\left(\%\right)$$ (3)

Where “Wo” and “Wd” are the weights of dried hydrogels before and after polymer extraction, respectively.

### 2.4 Solid state characterization

Fourier transform infrared spectrometry (FTIR) analysis was employed to identify functional groups and possible chemical interactions between drugs and hydrogels. All samples were scanned from 4,000 to 500 cm^−1^ on Bruker FTIR. Scanning electron microscopy (SEM) was used to analyze the surface morphology of blank, pure drug, and drug-loaded semi-IPN hydrogels. To analyze the thermal properties and stabilities of blank, pure drug, and drug-loaded hydrogels, differential scanning calorimetry (DSC) and thermogravimetric analysis (TGA) were simultaneously performed on the SDT Q600 Series Thermal Analysis system at 15°C–400 C with a heating rate of 10 C/min in the presence of nitrogen stream. XRD was employed for the analysis of the physical state of CTZ HCl and hydrogels. All samples were scanned over a range of 5°C–90°C (Ahmad et al., 2018; Zahra et al., 2021; Shoukat et al., 2022).

### 2.5 Drug loading

CTZ HCl was loaded in an optimized formulation by employing the disc diffusion method. In brief, hydrogel discs were soaked in 100 mL of 1% w/v solution of CTZ HCl in a USP phosphate buffer of pH 7.4. These hydrogel discs were removed from the buffer solution once equilibrium was achieved. Excess drug solution from the hydrogel surface was removed with blotting paper, and the hydrogel was dried to attain a constant weight.

### 2.6 Drug content

Different methods were adopted to evaluate drug loading in the optimized semi-IPN hydrogels. Firstly, using the weight variation method, drug content was estimated as follows (Ranjha et al., 2016).

Amount of loaded drug=Wd−Wo (4)

$$Drug\;loading\;\left(\%\right)=\left(Wd-Wo\right)/Wo\times 100$$ (5)

Where “W_o_” and W_d_ are the initial and final weights of dried discs before and after drug loading, respectively. Secondly, by the extraction procedure, drug content was estimated by putting the weighed quantity of CTZ HCl-loaded discs in PBS pH 7.4. Every time, 25 mL of fresh PBS was added until no drug was detected in the pooled buffer solution. The amount of drug was estimated from the calibration curve, which was previously constructed on the UV spectrophotometer at λ_max_ of 231 nm (Afzal et al., 2018).

### 2.7 Drug release

*In vitro* CTZ HCl release was monitored for 2 h at pH 1.2 (simulated gastric residence time) and in the USP buffer of pH 7.4 for 72 h in the USP type II apparatus at 37°C ± 0.5°C and 50 rpm (Afzal et al., 2018). At different time intervals, 5 mL of the sample was collected and replaced with a fresh buffer of a specified pH to maintain sink conditions. Then, the samples were analyzed at 231 nm on the UV spectrophotometer to estimate drug concentration (Khan et al., 2011). Afterward, cumulative drug release (Eq. 6) was evaluated, and the Korsmeyer Peppas model (Eq. 7) was applied to understand the mechanism of drug release.

% cumulative release=actual drug release/amount of drug loaded∗100 (6)

$$Mt/Mo=k_{KP}\;t^{n}$$ (7)

Here, “Mt” refers to the amount of drug release at time “t”, “Mo” refers to the quantity of drug release at infinity, “K_KP_” is the release rate constant, and “n” is the diffusional coefficient.

### 2.8 Toxicity testing

We used the fixed-dose guideline number 420 for toxicity testing as set by The Organization for Economic Co-Operation and Development (OECD). All the protocols were approved by the Ethical Committee of GCUF, vide letter number GCUF/ERC/2153. Rabbits in the control group only received water and food, while the treated group received 2 g/kg of the optimized formulation (F5). All the physical changes, mortality rate, variation in body weight, and water and food consumption were monitored for 14 days. Blood samples were assessed for any changes in hematological and biochemical parameters. Afterward, the animals were sacrificed; their vital organs were weighed and fixed in a 10% v/v phosphate-buffered neutral formalin. Later, thin slices of their organs were stained with hematoxylin and eosin (H&E) for tissue analysis.

## 3 Results and discussion

**FIGURE 1:**
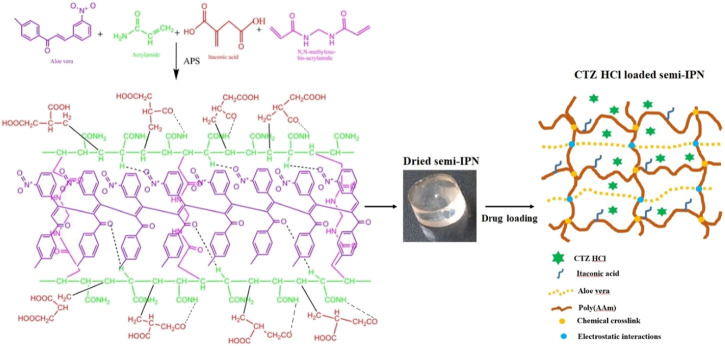
The graphical presentation of the initial ingredients, possible structure, and loading of CTZ HCl in IA-g-poly (AAm)/aloe vera semi-IPN hydrogel.

Initially, 10 different formulations were developed by varying the concentrations of aloe vera, followed by changing the concentrations of IA. The possible structure of a semi-IPN hydrogel is shown in Figure 1. Subsequently, swelling behavior and sol-gel contents were investigated to select an optimum formulation.

### 3.1 Swelling behavior

**FIGURE 2:**
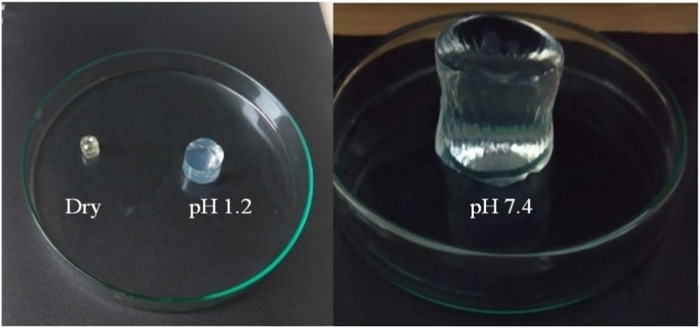
The optical images of dry and swollen hydrogels at pH 1.2 and 7.4.

To explore the influence of the pH of the media on the water absorption and swelling behavior, prepared semi-IPN hydrogels were immersed in 0.1 M HCl (pH 1.2) and a buffer solution of pH 7.4. Their appearance in the dry form and media of different pH are shown in Figure 2.

#### 3.1.1 Effect of aloe vera

**FIGURE 3:**
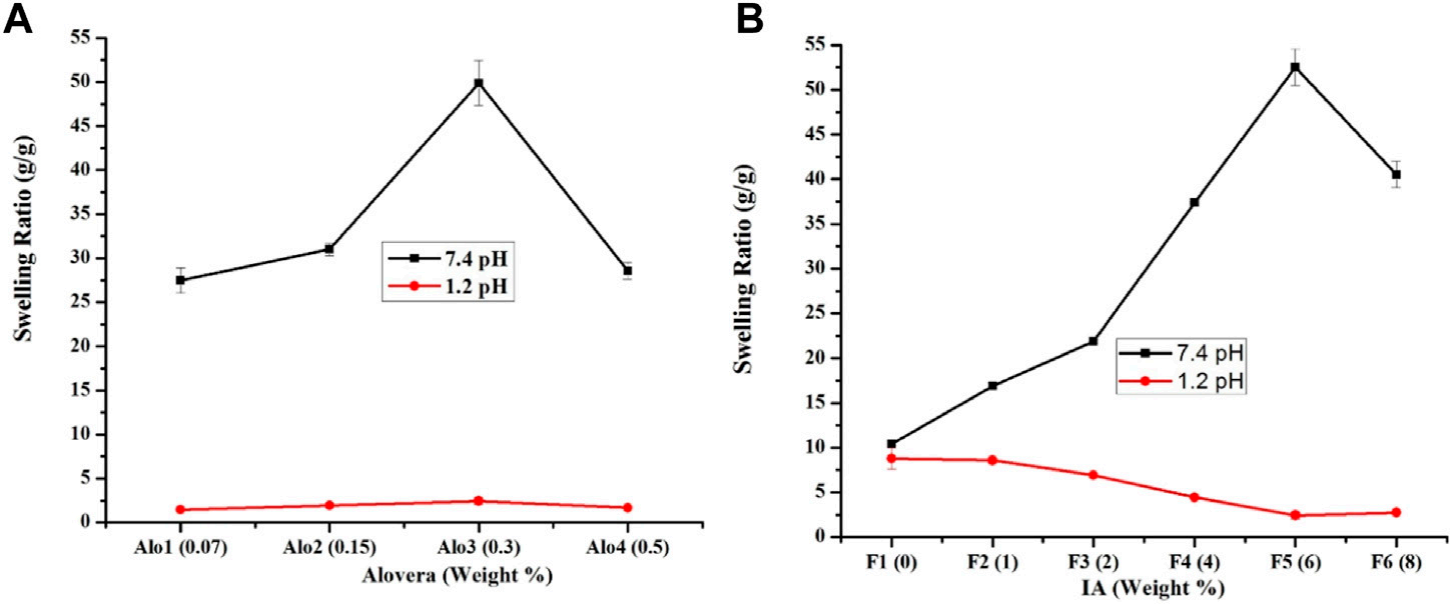
The effect of increasing concentrations of aloe vera **(A)** and IA **(B)** on the swelling ratio (g/g) of semi-IPN hydrogels.

Hydrogels immersed in acidic media presented the lowest water absorption owing to the protonation of the carboxylic groups. Here, the pH of the media was below the pKa of the ionizable groups of the aloe vera, resulting in a low swelling behavior. Contrarily, hydrogels immersed in a buffer of pH 7.4 exhibited higher water absorption and swelling, as shown in Figure 3A. In this solution, the carboxylic groups of the aloe vera are ionized (COO−) to increase the electrostatic repulsion between adjacent polymeric chains of the network and thus improve the swelling of the hydrogel.

Furthermore, increasing the concentration of aloe vera in various formulations significantly increased the swelling index of hydrogels. This behavior was attributed to the hydrophilic character of aloe vera. In this study, the hydrophilicity of the network increased proportionally with the concentration of aloe vera (Pereira et al., 2013). According to existing literature, hydrogels with higher aloe vera contents exhibited rapid absorption of water when immersed in a buffer solution of pH 7.4. The authors reported that the degree of swelling was enhanced with higher contents of aloe vera (Park and Nho, 2003). It is a well-known fact that highly crosslinked structures are not able to accommodate a high quantity of fluid within their network. However, the addition of aloe vera loosens the polymer network and increases the fluid uptake capacity (Bialik-Wąs et al., 2020). We further observed that on increasing the aloe vera contents above 0.3% w/w, swelling decreased (Figure 3A). It could be due to (a) increased crosslinking density and (b) a higher concentration; aloe vera might have occupied interstitial spaces and limited the interaction of crosslinked network with the media (Ajaz et al., 2022). After analysis, the formulation with 0.3 %w/w aloe vera (Alo3) was chosen for further testing.

#### 3.1.2 Effect of IA

The formulation with the optimum concentration of aloe vera was further modified with various concentrations of IA (0–8%w/w). Prepared hydrogels showed similar swelling behavior as observed for aloe vera, i.e., a low swelling at pH 1.2 and a higher swelling at pH 7.4 (Figure 3B). At a low pH, the swelling of all formulations decreased, while at a high pH, swelling increased with an increase in the concentration of IA up to 6%w/w. At a higher pH, IA might have contributed to higher concentrations of–COO^−^ ions along with free H+ ions within the hydrogel network. This could have increased the electrostatic repulsion between adjacent polymeric chains and osmotic pressure within the hydrogel to increase the swelling. Afterward, swelling decreased, as shown in Figure 3B, which may be due to the development of a dense polymeric network where swelling and penetration of dissolution media were difficult. Our observations are consistent with previous studies where swelling of hydrogel increased with an increase in the concentration of pH-sensitive monomers up to a certain concentration and then decreased afterward (Ajaz et al., 2021; Rehman et al., 2021).

### 3.2 Sol-gel fraction

The sol-gel fraction of hydrogels was used to estimate the non-crosslinked fraction of monomers or polymers. On increasing the IA concentrations, gel% increased while sol% decreased, as shown in Table 2. Here, upon increasing the concentration of the monomer, there was a higher crosslinking that ultimately enhanced the gel percent of hydrogels. Previous studies presented similar observations where IA-g-poly (acrylamide)/sterculia gum semi-IPN showed higher gel % with increasing content of monomers (Rehman et al., 2021). Based on swelling and gel content, F5 was chosen as the optimum formulation for further testing and evaluation.

**TABLE 2:** Effect of IA on the sol-gel fraction of semi-IPN hydrogels.

Code	IA (%w/w)	Sol%	Gel%	Standard deviation
F1	0	16.9	83.1	± 1.80
F2	1	13.5	86.5	± 2.06
F3	2	10.8	89.2	± 1.86
F4	4	9.4	90.6	± 2.00
F5	6	8.33	91.7	± 1.90
F6	8	7.95	92.1	± 1.26

All values are expressed as mean ± SD (*n* = 3).

### 3.3 FTIR

**FIGURE 4:**
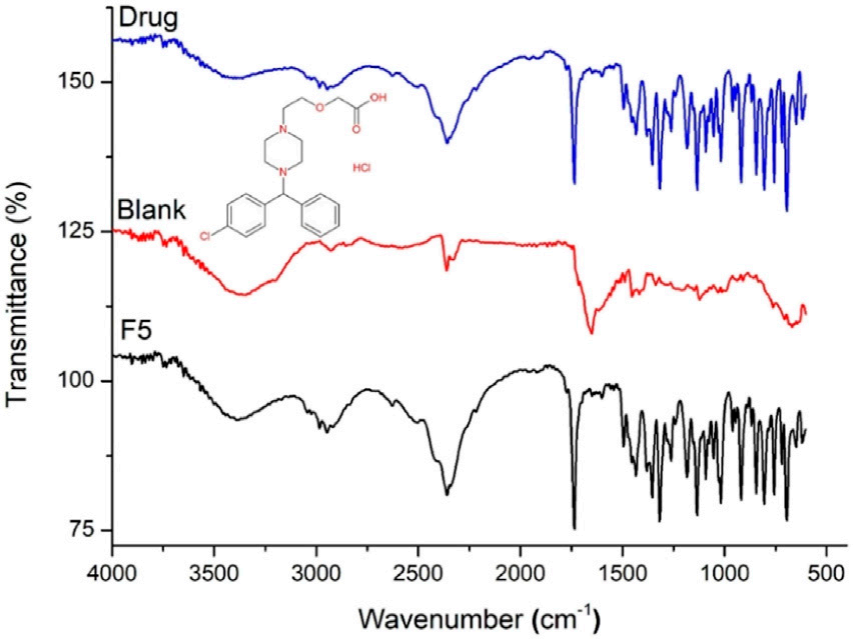
FTIR spectra of pure drug, blank, and drug-loaded semi-IPN hydrogels (F5).

FTIR analysis was used to evaluate any possible drug excipient interactions and to elucidate the network structure. We obtained and analyzed the FTIR spectra of blank, drug-loaded semi-IPN hydrogels, and CTZ HCl (Figure 4). In literature, aloe vera showed absorption bands at 3,390, 2,931, 1719, 1,407, and 1,254 cm^−1^ that represent -OH group, -CH vibration, -CO linkages, -CH_3_ vibration, and -C O C groups, respectively (Pereira et al., 2013). IA exhibited hydroxyl group (-OH) peak at 3,432 cm^−1^, -carbonyl (-C=O) stretch of -COOH group at 1,702 cm^−1^, and -C=C stretching at 1,631 cm^-1^. Whereas, AAm demonstrated -CH group stretching at 3,102, 3,034, and 2,813 cm^−1^, -HN stretching at 3,347 and 3,191 cm^−1^, and -C = C peak at 1,648 cm^−1^ (Rehman et al., 2021).

The blank hydrogel spectrum showed characteristic bands at 3,300 cm^−1^ due to the presence of amino and hydroxyl groups of aloe vera. A shift of peak from 1,425 to 1,417 cm^−1^ could be due to the incorporation of aloe vera as observed previously (Pereira et al., 2013). Absorption bands in the range of 3,427 to 1,636 cm^−1^ were attributed to the -OH stretching and -COO. These results for absorption bands are in agreement with previous reports (Pereira et al., 2011). Moreover, the absence of -C = C peaks of Aam and IA at 1,648 and 1,631 cm^−1^, respectively, indicated the involvement of monomers in the polymerization and grafting of IA onto the polymeric network.

FTIR spectrum of the pure drug showed intense peaks at 757, 1,024, 1,191, and 1,317 cm^−1^ corresponding to aliphatic chloro compound, alkyl substituted ether, the tertiary amine of piperazine ring of CTZ HCl, and–COOH groups, respectively (Sharma et al., 2014b; Sharma et al., 2015). CTZ HCl peaks remained intact in F5, which indicates the compatibility of the drug with the developed hydrogel.

### 3.4 SEM

**FIGURE 5:**
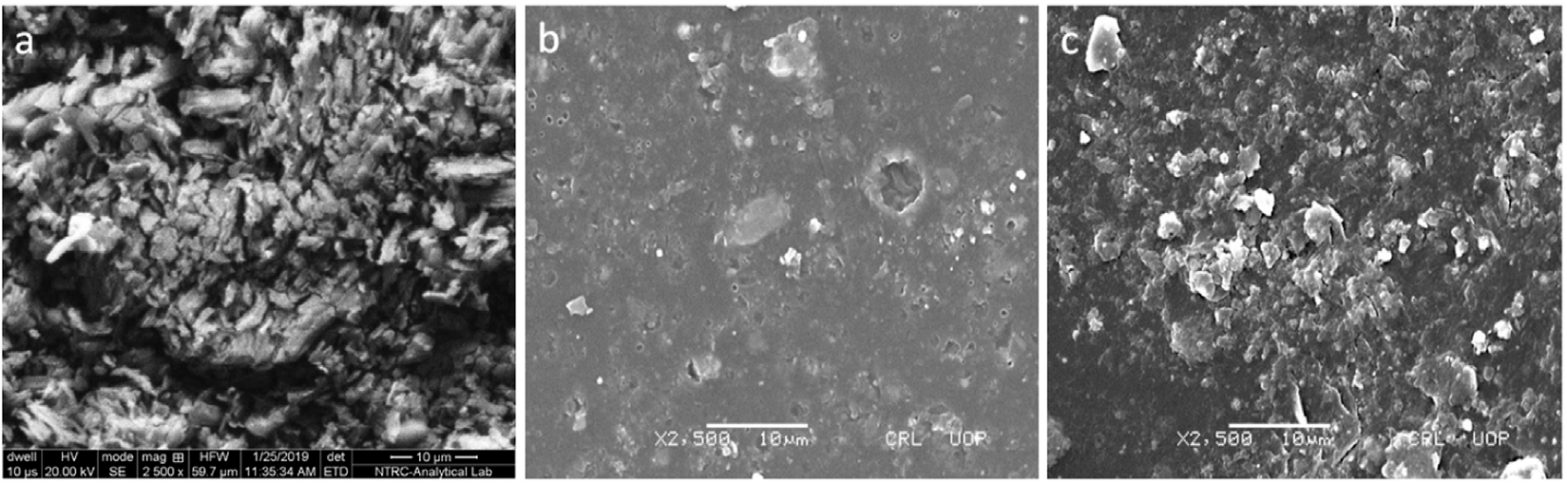
The SEM micrograph of **(A)** CTZ HCl, **(B)** blank, and **(C)** drug-loaded semi-IPN hydrogels (F5).

Surface morphology CTZ HCl and drug-loaded and blank semi-IPN hydrogels were analyzed by SEM micrographs. CTZ HCl showed an acicular shape with a smooth surface, partially agglomerated in bundles (Figure 5A). These findings are in agreement with previous observations about the crystalline nature of CTZ HCl (Douroumis et al., 2011). The SEM image of blank hydrogels depicted slightly rough and porous surfaces (Figure 5B), which might be due to the collapse of the polymeric network during drying. These surface pores of the hydrogel network could serve as the entry point for dissolution media. Furthermore, the porous assembly also contributed to a higher swelling ratio of hydrogels. Similar porous surfaces were observed in previous studies (Khalid et al., 2018). On the other hand, F5 showed a slightly rough surface with blocked pores and surface-adhered white particles (Figure 5C). These particles could be drug molecules that migrated to the surface along with dissolution media during drying after drug loading. This also indicates that the drug was uniformly distributed in the hydrogels. Furthermore, the presence of drug particles on the hydrogel surface could contribute to early drug release as observed by other researchers (Jabbar et al., 2021).

### 3.5 DSC

**FIGURE 6:**
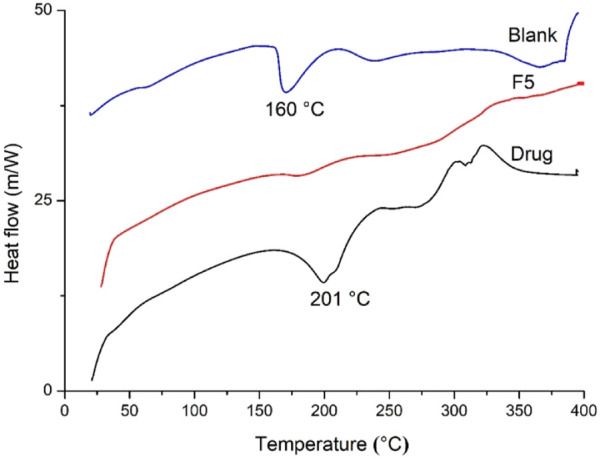
DSC thermograms of blank, F5, and blank semi-IPN hydrogels.

The DSC thermogram of blank hydrogels showed a melting peak of IA at approximately 160°C (Figure 6) as reported previously (Kirimura et al., 2011) and poly (acrylamide) above 300°C. Pure CTZ HCl exhibited a melting peak at approximately 201.3°C, followed by a degradation peak at approximately 220°C and is in agreement with previous studies (Katewongsa et al., 2017; Afzal et al., 2018). CTZ HCl peaks diminished in drug-loaded hydrogels (F5) indicating dispersion of the drug in an amorphous form. These results were further confirmed by XRD analysis.

### 3.6 TGA

**FIGURE 7:**
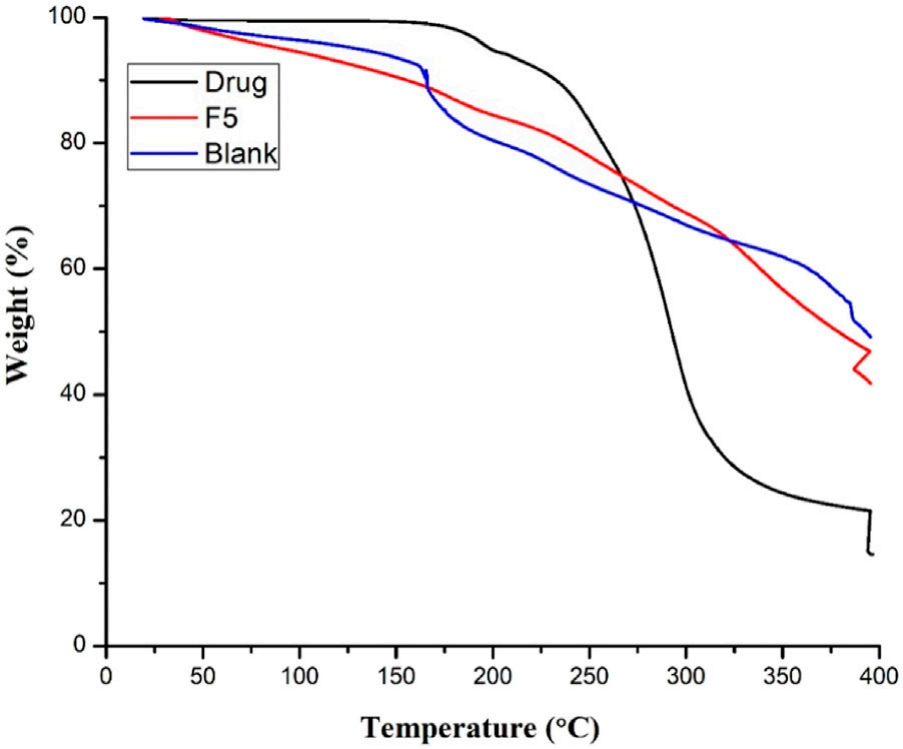
TGA thermograms of pure drug, blank, and CTZ HCl-loaded semi-IPN hydrogels.

TGA thermograms of CTZ HCl and blank and drug-loaded hydrogels are shown in Figure 7, which depicts the impact of thermal variation. The thermal degradation of blank hydrogel was accomplished in two steps. First, weight loss (8%) was observed up to 165°C which could be due to the loss of water molecules. Second, weight loss (42%) was detected between 165°C–00°C due to the degradation of IA chains of the polymer. Similar findings were observed elsewhere (Aghamohamadi et al., 2019) for aloe vera poly (vinylpyrrolidone) or aloe vera acetate poly (vinylpyrrolidone) electrospun fibers. In another study, IA-grafted-poly (AAm)/carbopol semi-IPN networks showed degradation of IA side chains from 165 °C onwards (Ajaz et al., 2021). Pure CTZ HCl showed ~ 5% weight loss up to 165°C. This weight loss increased (~92.8%) with the increase in temperature between 247°C–400°C, which corresponds to the thermal degradation of the drug (Sovizi and Hosseini, 2013). In comparison, the drug-loaded hydrogel presented slightly higher thermal stability when compared to the blank hydrogel. First, weight loss (11%) was observed up to 165°C, while 42% loss of weight was observed from 165°C–400°C. Thus, we can conclude that the prepared formulation was stable over a wide range of temperatures. Moreover, the incorporation of the drug did not affect the stability.

### 3.7 XRD

**FIGURE 8:**
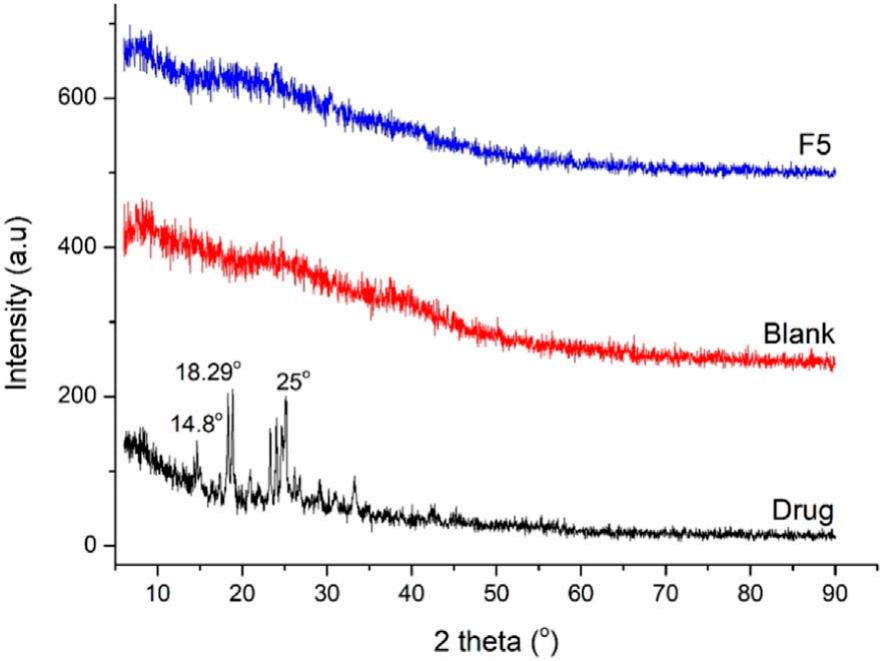
XRD of pure drug, blank, and CTZ HCl-loaded hydrogel (F5).

XRD analysis of CTZ HCl, blank, and drug-loaded hydrogel was carried out to establish the crystalline or amorphous nature. CTZ HCl presented sharp peaks at 8.3°C, 14.8°C, 18.29°C, 18.79°C, 23.97°C, 24.5°C, and 25°C (Figure 8), which indicate crystalline nature. In this literature, it is mentioned that IA is of a crystalline nature with peaks at 18°C, 22°C, 24°C, 30°C, 31°C, 36°C, 39°C, 44°C, 55°C, and 56°C (Sabaa et al., 2017), while poly (AAm) (Kumar et al., 2019) and aloe vera (Gontijo et al., 2013) are amorphous in nature. The intense peaks of CTZ HCl were absent in drug-loaded hydrogels, which indicates the dispersion of the drug at a molecular level. Furthermore, blank and optimized formulations (F5) were comparable. Both displayed broad and dull peaks, indicating the amorphous nature of hydrogels. Such amorphous networks also facilitate water uptake, swelling, and release of API.

### 3.8 Drug content

CTZ HCl was loaded in the optimum formulation (F5) that displayed maximum swelling at pH 7.4. Drug loading was determined by weight variation and the extraction process (Table 3). Similar patterns of CTZ HCl loading were also observed for gelatin/sodium alginate-based hydrogels (Ranjha et al., 2016).

**TABLE 3:** Drug loading (% g/g) in the optimized formulation.

Method	Drug loading (% g/g)	Standard deviation
Weight variation	28.1	±0.03657
Extraction	27.5	±0.00036

All values are expressed as mean ± SD (*n* = 3).

### 3.9 Drug release

**FIGURE 9:**
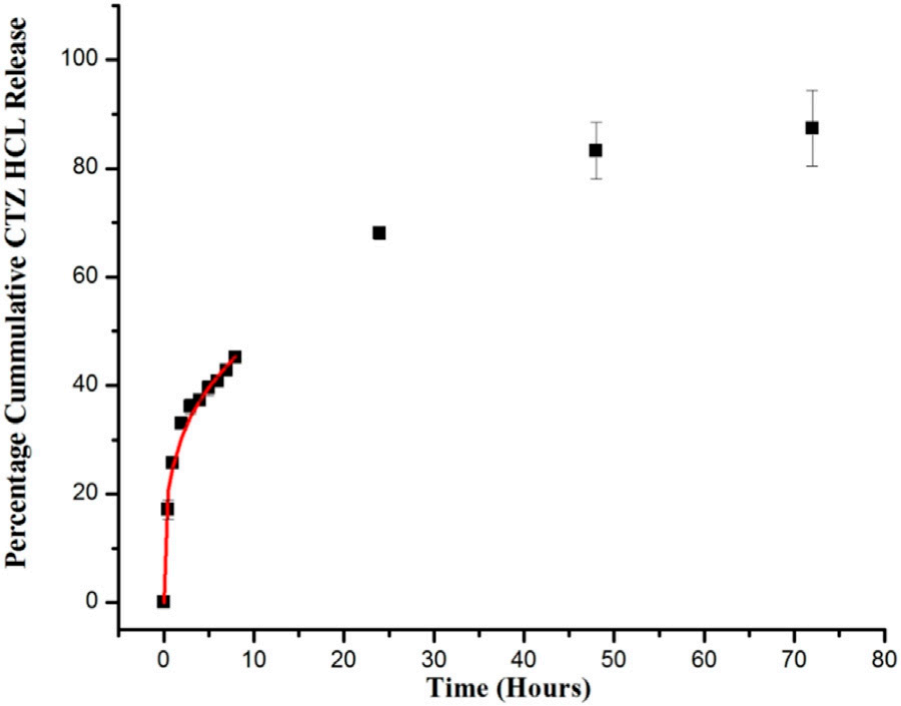
*In vitro* CTZ HCl release from semi-IPN hydrogel (F5). The release curve shows the Korsmeyer Peppas model on the first 60% release (Shown by the red line).

For developed hydrogels, cumulative % CTZ HCl release was very poor at pH 1.2, which was due to limited swelling of hydrogel in an acidic environment as mentioned in swelling results above. In acidic media, burst release of CTZ HCl (~32%) was observed during the first 2 hours of the study. This could be due to the presence of CTZ HCl molecules on the surface of hydrogels as verified by SEM images. Moreover, this initial burst release could also be due to high concentration gradients of the drug between the hydrogel exterior and release media, which could be the driving force for CTZ HCl diffusion. Thus, initial burst release was usually established as soon as the hydrogel was placed in the release media (Zhang et al., 2004). Afterward, as the pH of the media was switched to basic, the drug was released in a controlled manner (~86%) owing to the swelling of the three-dimensional network, which released the deeply-seated drug (Figure 9). At this pH, -COOH groups of the semi-IPN network were ionized, thus causing the electrostatic repulsion of network chains and ingression of dissolution media that not only caused swelling of the network but also caused the dissolution of the drug for constant release at the desired location (Fallon et al., 2019; Ajaz et al., 2021). Hydrogels are generally designed to respond to various physiological changes and, accordingly, alter the release pattern of entrapped drugs. In literature, we have successfully demonstrated that our developed semi-IPN exhibited changes in its swelling behavior, network structure, and permeability with variations in pH to release the drug at the desired location. Existing literature shows that CTZ HCl from IA-g-poly (AAm)/sterculia gum (Rehman et al., 2021) and sulfasalazine loaded in pectin-g-(PEG-co-MAA) pH-sensitive semi-IPN network released trapped molecules at a colonic pH (Abbasi et al., 2019).

### 3.10 Drug release kinetics

The Korsmeyer Peppas model was used to elucidate the mechanism of CTZ HCl release from semi-IPN hydrogels. The correlation coefficient (*R* ^2^) value that was near “1” explains the suitability of the model. For the optimized formulation (F5), the “n” value was below 0.45, indicating the involvement of the Fickian diffusion mechanism (Table 4).

**TABLE 4:** Release kinetics of semi-IPN hydrogel (F5).

Model	Parameter	Value
Korsmeyer Peppas	The release rate constant (K)	24.969
	Diffusion exponent (n)	0.287
	Correlation coefficient (*R* ^2^)	0.9867

### 3.11 Toxicity testing

#### 3.11.1 The general condition of animals

During the course of the study, all animals in the control and treated groups were tested for different parameters. We did not observe any abnormal clinical findings (Tables 5, 6), indicating the non-toxic nature of the developed formulation.

**TABLE 5:** Clinical findings of control and treated group.

Observations	Control	Treated
Sign of illness	NA	NA
Dermal toxicity (flare and ulceration)	NA	NA
Ocular toxicity (salivation and redness)	NA	NA
Mortality	NA	NA
Body weight (kg)		
Pretreatment	1.62 g ± 0.16	1.89 g ± 0.31
Day 1	1.61 g ± 0.05	1.81 g ± 0.12
Day 7	1.59 g ± 0.53	1.83 g ± 0.81
Day 14	1.60 g ± 0.52	1.82 g ± 0.08
Water intake (mL/day)		
Pretreatment	170 mL ± 1.72	166 mL ± 1.72
Day 1	168 mL ± 1.72	153 mL ± 1.92
Day 7	162 mL ± 0.36	158 mL ± 0.91
Day 14	160 mL ± 1.62	159 mL ± 0.73
Food intake (g/day)		
Pretreatment	70 ± 1.55	69 ± 1.6
Day 1	71 ± 1.54	70 ± 1.7
Day 7	70 ± 1.15	69 ± 2.4
Day 14	69 ± 2.14	68 ± 1.1

All values are expressed as mean ± SD (*n* = 3).

**TABLE 6:** The effect of optimized semi-IPN hydrogel on weight (g) of vital organs.

Organ	Control	Treated
Heart	4.14 ± 0.15	3.92 ± 0.11
Liver	9.56 ± 0.22	8.63 ± 1.20
Lung	8.92 ± 2.01	9.31 ± 1.20
Kidney	7.30 ± 0.72	7.70 ± 1.60
Stomach	12.63 ± 0.51	13.03 ± 0.51

All values are expressed as mean ± SD (*n* = 3).

#### 3.11.2 Blood analysis

Blood samples of control and tested animals were analyzed to evaluate hematological and biochemical parameters, as shown in Tables 7, 8, respectively. These parameters show that the optimized formulation did not affect the composition of blood as all the values were within the normal range. Moreover, the livers and kidneys were also working normally. This suggests the nontoxicity of the developed formulation.

**TABLE 7:** Hematology of control and treated groups.

Hematology	Control	Treated	Normal values
Hb (g/dL)	15.9 ± 0.45	16.2 ± 0.17	14–18
pH	7.14 ± 3.08	7.2 ± 0.42	7.3
WBCs × 10^9^/L	16.3 ± 1.16	13.9 ± 0.61	4–17
RBCs × 10^12^/L	8.92 ± 0.05	5.87 ± 4.12	4.5–6
Platelets × 10^9^/L	388 ± 1.53	389 ± 0.81	150–400
Monocytes (%)	2 ± 0.52	5 ± 1.08	2–11
Neutrophils (%)	41 ± 0.84	42 ± 1.81	35–65
Lymphocytes (%)	48 ± 1.72	57 ± 1.72	23–53
MCHC (%)	33.8 ± 1.62	33.4 ± 0.73	32–36

All values are stated as mean ± SD (*n* = 3).

**TABLE 8:** Liver and kidney profile of control and treated group.

Biochemical analysis	Control	Treated	Normal values
Bilirubin	0.79 ± 0.45	0.86 ± 0.17	0.2–1.2 mg/dL
AST	46 ± 1.08	49 ± 0.42	10–35 µ/L
ALT	39 ± 0.16	41 ± 0.61	9–43 µ/L
Alkaline phosphatase	289 ± 0.05	292 ± 0.12	65–306 µ/L
Blood urea	25 ± 1.53	27 ± 1.81	10–50 mg/dL
Serum creatinine	0.9 ± 0.52	0.81 ± 0.08	0.6–1.2 mg/dL
Serum uric acid	3.6 ± 0.84	3.8 ± 1.81	3.4–7.1 mg/dL

ALT: alanine aminotransferase, AST: aspartate aminotransferase. All values are expressed as mean ± SD (*n* = 3).

#### 3.11.3 Histology

**FIGURE 10:**
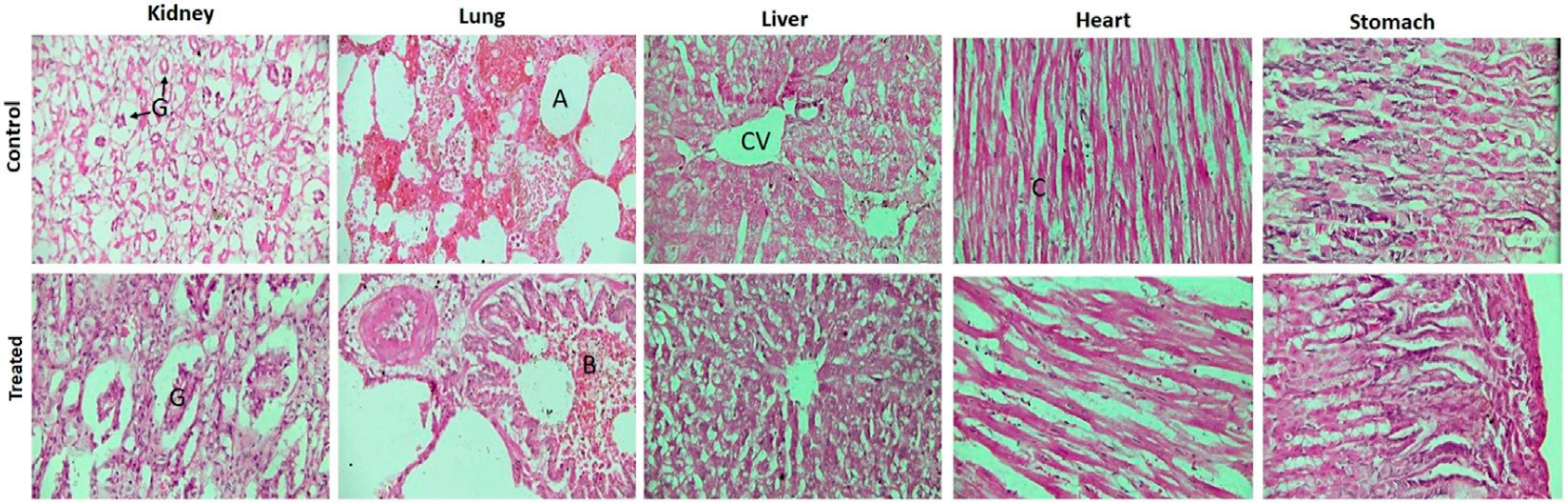
Histopathological images of vital organs. All images were acquired at 40x on a Tinocular microscope, Accu-Scope 3,001 fitted with a five-megapixel camera. Here, the alphabets represent, G: glomerulus, B: bronchiole, A: alveolus, CV: central vein, and C: cardiac muscles.

After H&E staining of micro sections of vital organs of both groups, slices were observed under the microscope to detect any possible changes in the cellular structure. In comparison, we did not observe any significant changes in the histology between the treated and control groups (Figure 10).

The kidneys of both groups showed intact glomeruli without any signs of necrosis and bleeding. The lungs also presented a normal structure, i.e., bronchiole, alveolus, and blood vessels without any signs of infiltrations, edema, and inflammation. In the liver, hepatocytes were arranged around the central vein. The heart displayed branched-striated myofibers arranged normally without any signs of hemorrhage. The histology of the stomach also presented normal cellular structure, intact mucosa without any signs of ulcer, and any other gastropathy. These findings are consistent with previous findings (Rehman et al., 2021; Ajaz et al., 2022). These findings suggest the biocompatibility and non-toxicity of semi-IPN hydrogels, identifying them as a suitable carrier for use in humans.

## 4 Conclusion

IA-g-poly (AAm)/aloe vera semi-IPN hydrogels were prepared and characterized by various *in vitro* and *in vivo* tests. The swelling of hydrogels was low at acidic pH but increased at higher pH (7.4). Formulation F5 with 0.3% w/w aloe vera, 6% w/w IA, and 20% w/w AAm was considered optimum, owing to better swelling characteristics. The solid-state characterization of the optimized hydrogel confirmed the presence of CTZ HCl in an amorphous form without any interaction with prepared semi-IPN hydrogel. The optimized formulation released limited amounts of the drug at a low pH, while it released maximum amounts of the entrapped drug in a controlled manner at pH 7.4. Hydrogels did not show any toxic effects on vital organs and blood components, and the function of the kidney and liver remained normal. Thus, prepared semi-IPN hydrogels can be deemed as a safe and effective carrier for the targeted delivery of CTZ HCl.

## Data Availability

The raw data supporting the conclusion of this article will be made available by the authors, without undue reservation.
